# User-Experience with Haptic Feedback Technologies and Text Input in Interactive Multimedia Devices

**DOI:** 10.3390/s20185316

**Published:** 2020-09-17

**Authors:** Bruno Silva, Hugo Costelha, Luis C. Bento, Marcio Barata, Pedro Assuncao

**Affiliations:** 1 School of Technology and Management, Polytechnic of Leiria, 2411-901 Leiria, Portugal; bruno.s.silva@ipleiria.pt (B.S.); hugo.costelha@ipleiria.pt (H.C.); luis.conde@ipleiria.pt (L.C.B.); 2 Institute for Systems Engineering and Computers, 2411-901 Leiria, Portugal; 3 Institute for Systems and Robotics, 3030-290 Coimbra, Portugal; 4 Tech4Home, 3700-121 S. João da Madeira, Portugal; marcio.barata@tech4home.pt; 5 Instituto de Telecomunicações, 2411-901 Leiria, Portugal

**Keywords:** remote control devices, user experience, haptic feedback, text input methods, smart TV

## Abstract

Remote control devices are commonly used for interaction with multimedia equipment and applications (e.g., smart TVs, gaming, etc.). To improve conventional keypad-based technologies, haptic feedback and user input capabilities are being developed for enhancing the UX and providing advanced functionalities in remote control devices. Although the sensation provided by haptic feedback is similar to mechanical push buttons, the former offers much greater flexibility, due to the possibility of dynamically choosing different mechanical effects and associating different functions to each of them. However, selecting the best haptic feedback effects among the wide variety that is currently enabled by recent technologies, remains a challenge for design engineers aiming to optimise the UX. Rich interaction further requires text input capability, which greatly influences the UX. This work is a contribution towards UX evaluation of remote control devices with haptic feedback and text input. A user evaluation study of a wide variety of haptic feedback effects and text input methods is presented, considering different technologies and different number of actuators on a device. The user preferences, given by subjective evaluation scores, demonstrate that haptic feedback has undoubtedly a positive impact on the UX. Moreover, it is also shown that different levels of UX are obtained, according to the technological characteristics of the haptic actuators and how many of them are used on the device.

## 1. Introduction

The processing capabilities of a Smart TV and internet connectivity have definitely changed traditional TV systems, providing rich user environments enabled by a wide range of interaction possibilities [1,2]. The paradigm of multimedia consumption is changing, moving from traditional passive content viewing to interactive forms. Enabled by services on demand and applications that support multiple connected systems (games, social networks, internet browsing, etc), the user has an increasing active participation, adapting and interacting with multimedia content. This new paradigm has strong impact on the UX, leading to recent research on the quality and usability of interaction technologies [3,4,5]. Conventional TV RCD use mechanical buttons as their main technology for interacting with the user, although this technology provides a limited UX. As a consequence, the interaction between devices and controlled equipment has been evolving towards the adoption of new HCI technologies [6,7], including both mechanical elements, sensors and actuators. Rasteiro et al. studied the RCD improvement in terms of their UX, due to the introduction of an absolute navigation functionality, capable of autonomously, and with good accuracy, computing the 3D absolute orientation of the RCD and controlling a 2D pointer position on a TV screen [8]. The absolute 2D navigation functionality revealed a great improvement in UX when compared to the relative 2D navigation functionality, commonly referred to as airmouse. The advantages of using feedback when typing in mobiles devices were also demonstrated in an experimental research study, addressing the effect of feedback on chording keyboards [9]. This study provides relevant results and analyze the influence of having visual, audio or no feedback at all on the typing process.

In recent years, other technologies that have been under fast research and development, became popular in smartphones and are progressively being used to enhance the functionalities of RCDs with the purpose of improving the UX. For instance, haptic feedback and touch interfaces are increasingly being used in HCI devices to control Smart TVs. Common touch interfaces include individual buttons, slider bars or single touch trackpad surfaces. Haptic feedback virtues as a useful mechanism for HCI were first recognized in the mid-nineties [10], with recent haptic feedback being used in interfaces specifically targeted for visually-impaired people [11], medical training [12] and virtual reality industrial applications [13], to improve the UX in these application areas.

Although touch interfaces in RCD are nowadays ubiquitous in consumer electronics, they still suffer from major limitations on the UX, namely the lack of haptic feedback [14,15], and the absence of both user-friendly text input/editing methods and advanced controls for multimedia equipment and content [16,17,18,19]. This is particularly true considering that most interaction with multimedia systems, such as Smart TVs, require that the user is looking at the TV screen, preventing him/her to look at the RCD. Current haptic feedback technologies allow the same actuator to generate different touch feedback sensations, by changing the actuation/braking times and frequencies. These different sensations, provided to users, are known as haptic feedback effects. By varying the control signal (actuation/braking time and frequency) applied to an ERM and an LRA, Silva et al. identified which type of actuator and feedback effects provide the best UX [20]. Advanced interaction with smart TVs may be enhanced by the introduction of two interaction concepts: multi-touch input interface and multi-level input sensing, such as piezoelectric sensors or FSR, which open the possibility of using various control levels [21]. On the other hand, PCAP input surfaces can accurately detect multiple simultaneous touches with high spatial and temporal resolutions [22].

This research work is a contribution to improve the design and implementation of remote control devices, presenting a subjective evaluation study of state-of-the-art emerging interface technologies, combined with haptic feedback solutions. The UX is evaluated in regard to seven interface methods and four types of haptic feedback technologies that can be incorporated in advanced remote control devices. The interface method includes conventional mechanical buttons and advanced interfaces such as, airmouse, single touch trackpad, slider bar, punctual touch button, multi-level sensing button and multi-touch QWERTY keyboard. The research also focus on state-of-the-art technologies to generate haptic feedback, particularly the ones that can be used in small battery-powered remote control devices, namely ERM, LRA and PEA. These type of actuators are used to implement buttons with haptic feedback, and they are studied in comparison with the conventional haptic feedback technology used in RCD, i.e., mechanical buttons.

## 2. Haptic Interface and Feedback Technologies

Human interaction with different types of multimedia equipment and applications is mostly achieved via single-touch binary interfaces, through conventional mechanical buttons. In most cases the target equipment is easier to control if, besides haptic feedback, it also includes multi-level and single/multi-touch control.

An FSR sensor can output a signal that is proportional to the force being applied by the user’s finger, with the value of its resistance varying according to the applied force. These sensors consist of a conductive polymer with such electrical characteristic, i.e., its resistance changes according to the force applied to its surface. Compared to strain gauges, this type of sensor is much simpler to use, not requiring Wheatstone bridges to linearize the output signal. When no force is being applied to these sensors, they behave as an open circuit, meaning that there is no current consumption, therefore not compromising autonomy. Calibration of the measurement system is also unnecessary, since the focus will be on the relative measured value rather than the absolute one.

Punctual touch buttons can be implemented through the use of capacitive buttons, which allow the implementation of buttons without moving parts and can even work without direct contact. Approaching a finger to a capacitive button causes a variation in the sensor capacity in relation to the ground. This technique is sensitive to parasitic capacities between the capacitive buttons and power/ground planes, conductors and other circuit components. To minimize the parasitic capacities, guard rings are usually placed around each capacitive button with the same voltage as these, ensuring that there are no parasitic capacities resulting from potential differences between sensors and other circuit constituents.

**Figure 1:**
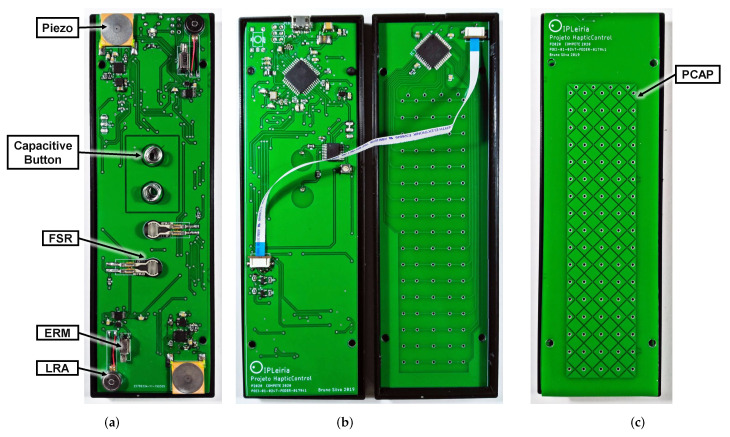
Developed prototype; (**a**) Top layer of module A; (**b**) Bottom layer of the module A (left) and top layer of module B (right); (**c**) Bottom layer of module B.

PCAP panels can detect multiple simultaneous touches with response times in the order of ms. The mutual capacity method is based on measuring the capacity between a pair of electrodes. Projected capacitive touch detection panels of mutual capacity, can be built with an array of X and Y electrodes, in two or four layers (see Figure 1c). This electrode configuration results in a projected electrostatic field over the sensor. When approaching a finger to the sensor formed by the X and Y electrodes, capacity coupling occurs between the finger and the electrodes, and the touch position can be determined. The size and number of electrodes to use depend on the size and resolution desired for the sensor. Furthermore, this technology allows the use of plastic-based surfaces, up to a few millimeters, between the sensor and the user fingers, a distance which is required in an RCD assembly.

There are three main types of actuators capable of generating diverse haptic feedback effects for handheld control devices used in interactive multimedia systems. Such actuators, known as ERM, LRA and PEA [23], are characterised by the underlying technology that is responsible for producing such effects. ERM actuators are mounted on the device and generate a mechanical vibration with a given frequency by spinning an eccentric mass attached to a small DC motor. Users can perceive different haptic feedback sensations from different vibration patterns that can be generated by using a pre-defined number of mass geometries with several possible axial orientation and/or positions. This is obtained by controlling the rotation speed of the DC motor and also the speed gradients through PWM control signals. If multiple ERM are combined together, then it is possible to generate more sophisticated vibration patterns, significantly increasing the range of possible effects. For instance, the effect of a mechanical button press can be realistically simulated by using combined feedback effects

The LRA is comprised of four main elements: a coil, a spring, a mass and a magnet. Mechanical vibrations are generated along an axis with the coil driving the mass. In comparison with ERM, this arrangement results in more limited vibration amplitude and frequency range. Nevertheless, since ERM operation is based on electromechanical commutation while LRA actuators are brushless, one advantage of the latter is that the only moving element prone to failure, are the springs. Therefore LRA actuators are in general of smaller size, more reliable and they also operate with higher energy efficiency, especially at their resonant frequency. Like the ERM, multiple LRA actuators can be combined to obtain more complex mechanical vibration effects.

Traditionally, PEA modules are based on one or more layers of ceramic materials, which expand or compress with the application of an electric potential. Applying a time-varying stress, the material vibrates with the frequency of the applied stress. These elements can be extremely thin (typically between 0.2 and 1.0 mm thick), allowing the application in smaller spaces. They are also characterized by a very short acting time, and a wide range of frequencies with which they can work, thus allowing higher dynamic range of effects than ERM and LRA, albeit with a lower vibration amplitude. They can also be more easily produced in different forms, when compared to ERMs and LRAs.

This heterogeneous sensor and actuator architecture that results from using quite diverse technologies as described above, requires a low level control hardware reasoning device, usually a microcontroller. In general, it is also required to handle HID, namely automatic identification of USB-based HID of interactive devices for Smart TV/Box.

## 3. User Experience Evaluation Setup and Methodology

This section describes the setup and methodology used to evaluate the UX for different types of haptic feedback effects and interactions with Smart Box/TV, using a remote control device. It starts by providing a description of the hardware and firmware specifically developed for this purpose, and then describes the overall test scenario and subjective evaluation methodology.

### 3.1. Test Setup

The test setup was built taking into account the overall goals of the UX evaluation and underlying experiments, which should allow to assess the following:

- Evaluate user opinions in single-click interactions, namely by comparing the use of traditional mechanical buttons with capacitive-based technologies using different haptic feedbacks;
- Evaluate user satisfaction perceived through interactive experiments, based on different dynamic inputs;
- Evaluate the UX when performing more complex interactions requiring text-based input.

In order to conduct these subjective evaluations, a prototype of a remote control device was specifically developed to evaluate the UX when using haptic feedback with different types of interactions. The prototype, shown in Figure 1, comprises two PCBs, each one with two-layers. We will refer to the PCB with more components, including sensors, actuators and a microcontroller, as module A (this is shown in Figure 1a,b, on the left), and the other as module B (shown in Figure 1b, on the right, and Figure 1c).

Given the evaluation tests performed in this research, it is of utmost importance to be able to implement a keyboard/mouse-like interaction with the Smart TV/Box. Therefore, in order to accelerate the development and implementation of the qualitative testing, the ATMEGA32U microcontroller was used, mainly due to its capability of being automatically identified as an USB-based HID. Relevant electrical parameters and modules configurations were defined in accordance to the components datasheets and communications standards, e.g., I2C communication frequency is 100 kHz. The decision not to use a battery and connect through an USB cable was also made in order to reduce the time needed to carry out the evaluation tests.

Two FSR were used for dynamic touch detection (see Figure 1a). The FSR used is the 5 mm diameter model from Interlink, FSR 400 short, which as a resistance ranging from 10 MΩ, when not pressed, to 2.5 kΩ when a 1.0 kg force is applied [24]. These sensors allow a more advanced interaction due to the possibility of using various control levels, rather than only binary outputs typically associated with mechanical buttons and capacitive buttons. This type of interaction enables advanced functionalities such as, for instance, increasing the sound level with a speed proportional to the force applied by the user. To measure the resistance variation value caused by the pressure exerted on the FSR, a voltage divider was implemented, directly connected to an analog port of the microcontroller.

Punctual touch buttons were implemented through capacitive buttons, with two capacitive sensors directly built into the top layer of module A, using a circular shape (see Figure 1a). The MTCH108 IC from Microchip [25] was used as the controller. This controller has a guard feature to minimize the parasitic capacitances between conductors, the sensor and the power/ground planes, as well other components. When such feature is enabled, up to seven capacitive sensors can be used with the same controller. In this work, guard rings were placed around each capacitive sensor and connected together to keep all of them at the same voltage. This is necessary in small devices, because most components are closely located.

A trackpad was implemented using PCAP (see Figure 1c), using the controller MTCH6301 IC from Microchip. This controller provides quite flexible features, due to the possibility of defining a variable number (up to a maximum of 10) of simultaneous touch and predefined gestures. In the case of applications in remote control devices, a particularly relevant characteristic is the possibility of using surfaces of different materials between the user fingers and the sensor itself. For instance, either a glass surface of up to 5 mm thick or a plastic surface of up to 3 mm thick, can be used. Note that such trackpad allows further functionalities beyond conventional mouse-type control, such as QWERTY touch-based Keyboard or slide bar. Regarding the mechanical buttons and airmouse, the tests were performed using the CY5672 PRoC BLE Remote Control Reference Design Kit [26]. This development kit also includes a trackpad, which is henceforth denominated CY-trackpad.

Nowadays, there are several ICs specifically designed for integration in different types of devices using haptic feedback functionalities. In the case of ERM actuators, one can find a wide range of configurations, from single-ended drivers with only one transistor to more efficient ones such as those using H-bridges. For LRA actuators, since it is necessary to generate the AC control signal from the main DC power source, some additional electronics is required for such purpose. There are also some ICs specifically designed to operate as drivers for haptic actuators, which can be used for both ERM and LRA. This is the case, for instance, of the Texas Instruments DRV2605L [27], which also includes an operational mode for detection of self-resonance, thus avoiding the need for presetting and calibration of different frequencies for each actuator, i.e., the IC itself can determine and memorize the resonant frequency of the LRA. This is a highly flexible controller, capable of generating one hundred and twenty three different haptic feedback effects from a royalty-free set provided by Immersion Corporation. Furthermore, in the case of ERM actuators, it is possible to control some parameters of each effect, such as intensity and acceleration/brake times. There are six different libraries with predefined parameters for the various effects. In regard to energy consumption, by comparing the LRA actuators with the ERM, in general one can find that the former can operate with less than 60% to 80% of energy than the latter [28]. For instance, using a 5 V voltage supply, a ~50 ms long, single click effect, consumes about 0.57 μAh using the LRA actuators, while it consumes 1.72 μAh using the ERM actuator. The PEAs require high peak voltages to vibrate, which can pose a problem for battery powered devices. However, there are already efficient solutions on the market which can generate these voltages using differential boosters and amplifiers. The DRV2667 IC from Texas Instruments [29] is one such case, allowing the control of PEAs.

**Figure 2:**
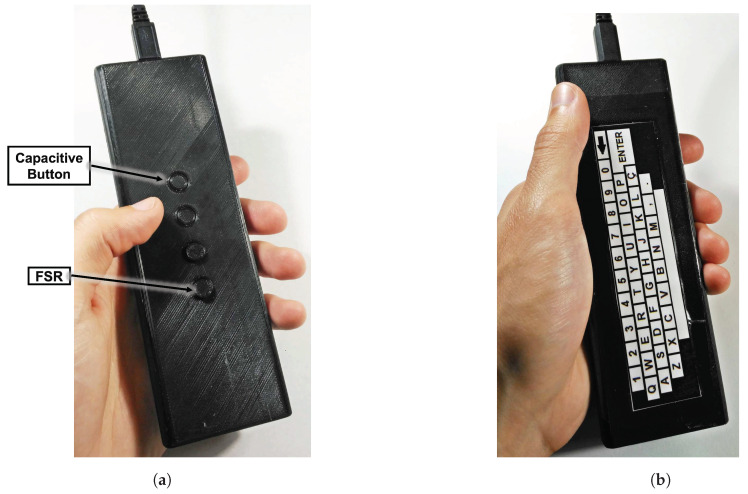
Evaluation prototype-3D printed structure; (**a**) Top outer part; (**b**) Bottom outer part.

The haptic feedback evaluation prototype includes the three types of actuators described earlier, namely the ERM (model Z30C1T8219731 from Jinlong Machinery Electronics, Inc., New York NY, USA), the LRA (model G0825001 from Jinlong Machinery & Electronics, Inc.) and the PEA (model Z63000Z2910Z1Z5 from TDK)-two of each are placed at the top part, while two other of each are placed at the bottom part, all on the top layer of module A, as shown in Figure 1a. The remote control device was connected to a PC through USB for the purpose of automatically acquiring and storing all the data obtained during the evaluation process. The experiment control and data acquisition was implemented using a python-based script running on the PC. To capture the keyboard input, we have used the Arduino standard keyboard library, which allows to identify the keyboard as an HID. For the prototype case, a 3D printed structured was built, with the buttons on the top part (associated with module A), while the bottom part (associated with module B) contained a flat surface with a QWERTY keyboard layout placed on it, as shown in Figure 2.

### 3.2. User Experience Evaluation Methodology

**Figure 3:**
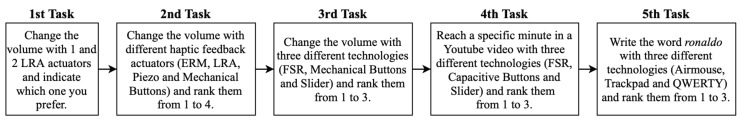
Test methodology.

Given the remote control prototype described above, a set of subjective evaluation tests was defined in order to assess the user experience in perceived in different interaction scenarios, using the various technologies presented earlier. As shown in Figure 3, the overall test procedure is divided in five tasks, each one having a specific goal, as described below.

In the 1st task, the goal was to gather the UX regarding the use of one versus two LRA actuators. The user was asked to increase and decrease the sound volume using the capacitive buttons, with the top button associated with one LRA-based haptic feedback, and the bottom button associated with the two LRA-based haptic feedback. In both cases, the haptic feedback effect associated with the LRA was the one preferred in previous studies, as detailed in [20]. Without knowing the difference between the two feedbacks, the user was asked to identify which effect she/he preferred.

The goal of the 2nd task was to compare the capacitive buttons with different haptic feedback and the mechanical buttons. In this task, the users were asked to change the sound volume in four use cases: (i) using a capacitive button with the ERM-based haptic feedback; (ii) using the capacitive buttons with the LRA-based haptic feedback; (iii) using the capacitive buttons with the PEA-based haptic feedback; (iv) using the traditional mechanical buttons. Only one actuator was used for each of the different technologies. For the ERM and LRA-based haptic feedbacks, the associated haptic feedback effect used was the one deemed preferred in previous experiments detailed in [20]. After testing the four different inputs with the associated haptic feedback, the users were asked to order each one according to their preference, from 4 (the one they liked most) to 1 (the one they liked least).

In the 3rd task, the underlying question was to evaluate whether there are some tasks where a dynamic, but yet simple, interaction might be preferable, such as changing the sound volume, changing the channel, or moving forward through a video. Therefore, the goal of the 3rd task was to evaluate the UX obtained by using a dynamic input with three different technologies, namely, an FSR, a mechanical button and a slider (implemented using the trackpad). In this task, the users had to change the sound volume in a more dynamic way. In the case of the FSR, the stronger the user presses the button, the faster the sound volume changes. In the case of the mechanical buttons, similar to controls used on Smart TVs, when leaving the finger pressing the button, the sound volume changes at a fixed speed. Both in the mechanical buttons and the FSR case, the users had two buttons, one for increasing the sound volume, and the other for decreasing it. In the slider case, there is an area in the trackpad with a bar drawn, with the sound volume change being proportional to the position of the user finger within that bar. In the end, the user had to score, in descending order of preference, each of the three use cases, assigning 3 to the one he/she liked most, and 1 to the one he/she liked least.

The goal of the 4th task was to evaluate precision dynamic input. In this case, moving forward to a particular time instant on a YouTube video, specifically at 2 min and 50 s (we choose YouTube given that it is a widely used video streaming platform, but any other video applications could have been used, given that the results and experience do not depend on the video application). In this task we compare the UX obtained by using the capacitive buttons (with forwarding velocity proportional to the time pressed), the FSR and a slider bar, already used in the 3rd task. However, the slider bar within the trackpad was 90º rotated in comparison with the previous task, in order to be horizontal, and thus coherent with the time bar shown on the video. Here, instead of increasing/decreasing the sound volume, the various inputs allow moving forward and backward in the video. The user had to score in descending order of preference each of the three cases, assigning 3 to the one he/she liked most, and 1 to the one he/she liked least.

Finally, the 5th task aimed to evaluate the various technologies for text-input. In this case, the users were asked to simulate a search action by writing the word ronaldo using three different technologies: an airmouse, a trackpad and a QWERTY keyboard. In this trackpad use case, the onscreen keyboard was shown on the monitor, with the keys being selected through the trackpad. In order to avoid long testing times, the CY-trackpad was used to test the trackpad use case. Regarding the QWERTY keyboard use case, we have used the developed trackpad-based module with the QWERTY layout placed on the top, as show in Figure 2b. In this case, we have obtained both a quantitative and a qualitative score of the user experience. Regarding the qualitative score, like in previous experiments, the user was asked to classify in descending order of preference each of the three cases, assigning 3 to the one he/she liked most, and 1 to the one he/she liked least. Regarding the quantitative score, the user was asked to press the space bar at the beginning and at the end of the test, and the amount of time spent by the user to input the desired word was measured. The diagram presented in Figure 3 shows the test methodology.

## 4. Experimental Results

**Figure 4:**
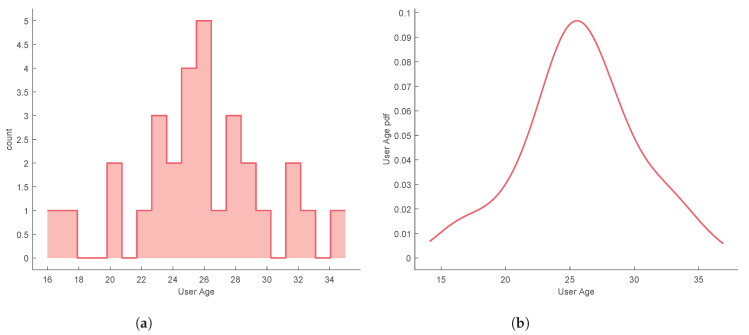
Users’ age characterization; (**a**) Age histogram; (**b**) Probability density function of users’ age.

A group of 30 users performed the subjective evaluation tests, consisting of 10 female and 20 male, aged between 16 and 36 years old, with different technological backgrounds. The user age distribution was non-uniform, having a mean age of 25.8 and a standard deviation of 4.4 years, as shown in Figure 4.

To familiarize the test users with the prototype and the tasks at hand, each user had some time (up to 10 min) to operate the RCD prototype and test each functionality before performing the evaluation tasks. The evaluation procedure consisted in each user performing each of the five tasks and answer the questions described in Section 3.2, regarding the quality of the UX. The testing environment consisted of a research Lab where the users were sit in front of a wide monitor.

**Figure 5:**
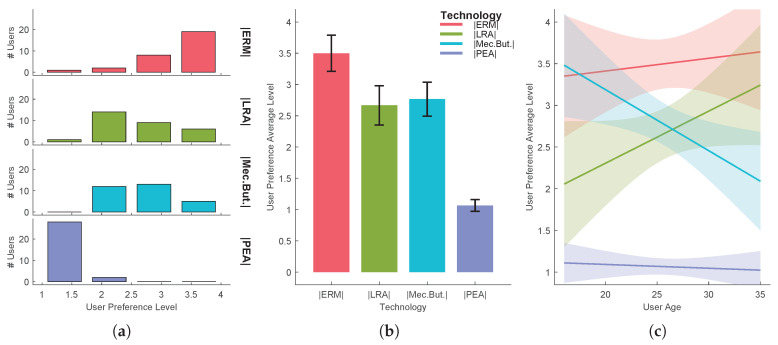
Task 2-user preference level in single-click actions using four different haptic technologies: (**a**) distribution per treatment, (**b**) mean and standard deviation, (**c**) evaluation function of users’ age.

**Figure 6:**
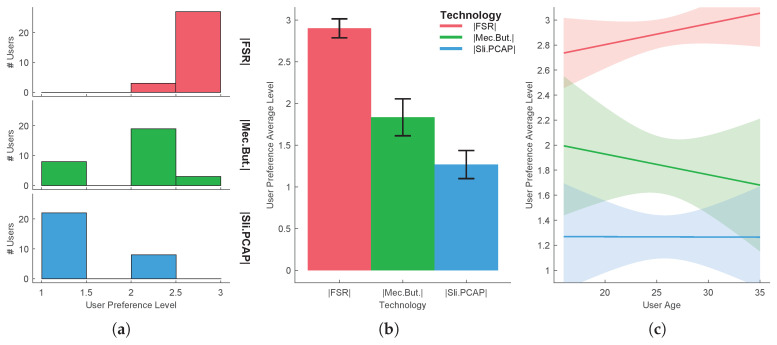
Task 3-user preference level in dynamic sound volume change using three different haptic technologies: (**a**) distribution per treatment, (**b**) mean and standard deviation, (**c**) evaluation function of users’ age.

**Figure 7:**
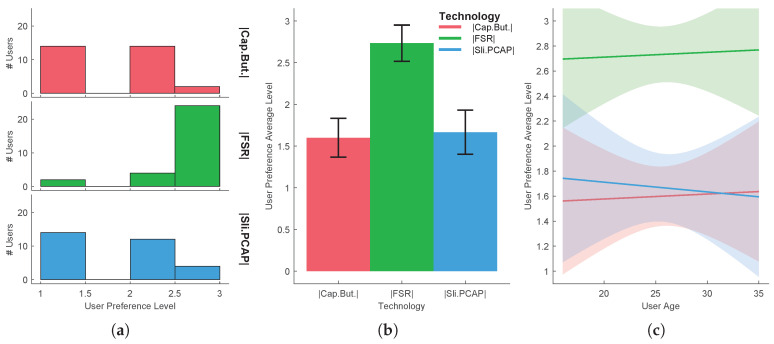
Task 4-moving through an YouTube video using three different haptic technologies: (**a**) distribution per treatment, (**b**) mean and standard deviation, (**c**) evaluation function of users’ age.

**Figure 8:**
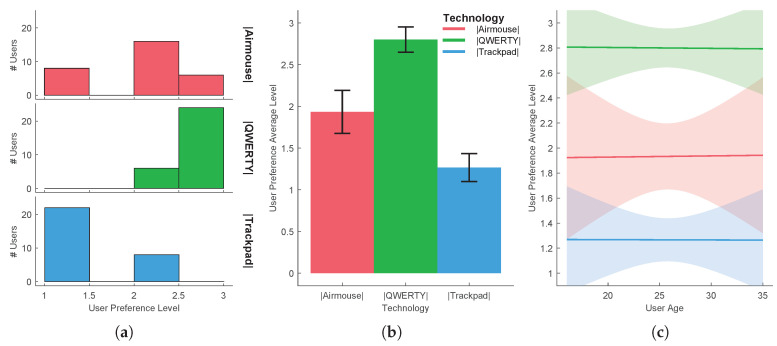
Task 5-text input using three different haptic technologies: (**a**) distribution per treatment, (**b**) mean and standard deviation, (**c**) evaluation function of users’ age.

**Figure 9:**
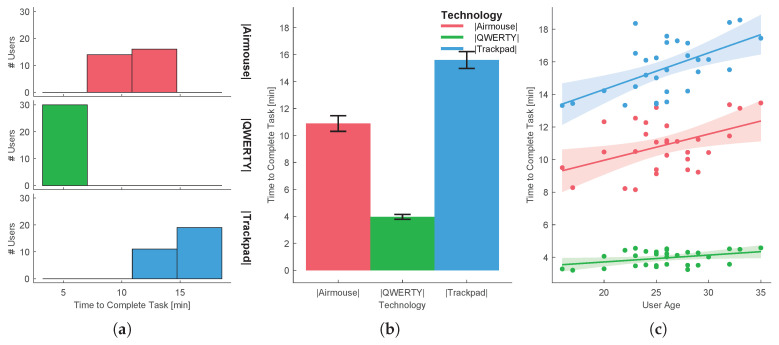
Task 5-time needed for typing the word “ronaldo” using three different haptic technologies: (**a**) distribution per treatment, (**b**) mean and standard deviation, (**c**) evaluation function of users’ age.

In order to reject the hypothesis that the experiment results come from a random chance in the sampling process, a hypothesis testing was performed for each task. The independent variable that varies between samples is called the factor. The different values or levels of the factor are called the treatments. Here the factor is the UX and the treatments are the several technologies/methods used. A common technique used for assessing statistical significance between the means of two or more independent treatments, is one-way ANOVA. One-way ANOVA is a parametric test, which requires the statistical distributions to be normal and homoscedastic. The Anderson-Darling test provides means to verify whether the data sample comes from a normal distribution. A rough evaluation of the treatment normality for the each sample data can be done by visually inspecting Figure 5a, Figure 6a, Figure 7a, Figure 8a and Figure 9a. When performing one-way ANOVA requirements validation, a common practice is to consider that the homogeneity of variances is not violated if the ratio of the largest and the smallest sample standard deviations is within 0.5 to 2, this ratio is henceforward called homogeneity ratio. Moreover Figure 5, Figure 6, Figure 7, Figure 8 and Figure 9 provide insight of how different are the variances for each treatment. A generalized linear fitting model to the data acquired in each experiment task is presented in Figure 5c, Figure 6c, Figure 7c, Figure 8c and Figure 9c, the bounding area display the fit results with 95% confidence. Figure 4, together with Figure 5c, Figure 6c, Figure 7c, Figure 8c and Figure 9c, provide the necessary information to assess UX of each technology per users’ age and the likelihood of the technology suitability for each targeted consumer’s age profile. Although one-way ANOVA is a robust technique, insensitive to small departures from normality and homogenity, these assumptions only hold if the sample sizes are large and equal for each group [30], which is not the case in this study. If the normality and homogenity condition do not hold, then a non-parametric test must be used. For non-parametric distributions, the rank version of one-way ANOVA was used, i.e., the Kruskal-Wallis test. The Kruskal-Wallis test, instead of numeric values, uses data ranks to compute the test statistics. The statistical significance test (one way ANOVA or Kruskal-Wallis) provides evidence, up to a significance level, that the treatments are not all equal. A post-hoc analysis must be applied to identify which treatments are significantly different. For parametric distributions, the Tukey HSD (Honestly Significant Difference) was used, while for non-parametric distributions it was used the Dunn & Sidak post-hoc analysis. All tests (Anderson-Darling, one-way ANOVA, Kruskal-Wallis, Tukey HSD and Dunn & Sidak) were made at significance level of D = 0.05. The well-known ANOVA table format was used to evaluate the Kruskal-Wallis significance test results. Such table format (e.g., Table 1) shows both the between-groups variation and within-groups variation, where the SS is the sum of squares, df is the degrees of freedom, MS is the mean squared error, F is the ratio between-groups mean square over within-groups mean square, and p is a probability. If the p-value is smaller than the significance level, then it indicates that at least one of the sample means is significantly different from the others. Moreover, the post-hoc test table format (e.g., Table 2) allows to assess whether there is a relevant difference between the two treatments, indicated as either YES or NO for the significance at 0.05.

*Task 1*

The 1st task assessment revealed that, for the haptic feedback of a single capacitive button click, the majority of the users, 97%, preferred the feedback produced by a single LRA actuator, when compared to using two LRA actuators.

*Task 2*

Regarding the 2nd task, users experienced four different technologies for a single-click interaction. Based only on average user preference level, one can conclude the following: users preferred the capacitive input sensor with the ERM-based haptic feedback with an average user preference level of 3.50, as shown in Figure 5. The second best classified option was the use of the mechanical buttons, with an average preference level of 2.77, but with a very close third option, using the capacitive input sensor with the LRA-based haptic feedback, with an average of 2.67 level. By far, the capacitive input sensor with the PEA haptic feedback was the least preferred one, with a user preference average level of 1.07. Following is an analysis that goes beyond the simple average user preference level assessment, i.e., analysis using significance tests. The Anderson-Darling test revealed that none of the treatment distribution was normal. The homogeneity ratio is 3.327, hence violating the homoscedastic requirement for a parametric analysis. Therefore the non-parametric Kruskal-Wallis test was applied to the data (see Table 1) followed by the Dunn & Sidak post-hoc analysis (see Table 2). There was a statistically significant difference between groups as determined by the Kruskal-Wallis test ($p=3.5362\times 10^{-16}$), and given the results of the Dunn & Sidak post-hoc analysis, except for the pair ERM-But.Mec. and LRA-But.Mec. there is a significant difference between all other treatments pairs. Since the ERM-based haptic feedback was identified as the preferred one and observing that, for the pairs ERM-But.Mec. and LRA-But.Mec., there is no significant difference, no conclusion can be made on the preferred interaction method.

*Task 3*

Regarding the dynamic sound volume change, tested in the 3rd task, the user preference average level is shown in Figure 6. Based only on average user preference level, one can conclude the following: the FSR-based approach had a 2.90 user preference average level, being the preferred one for most users. The second preferred option was the mechanical buttons, with 1.83, whereas the PCAP-based slider, with only 1.27 user preference average level, was the least preferred solution. Following is an analysis that goes beyond the simple average user preference level assessment, i.e., analysis using significance tests. Although the homoscedastic condition is not violated since the homogeneity ratio is 1.940, the Anderson-Darling test revealed that none of the treatment distribution was normal, hence violating the requirement for a parametric analysis. Therefore the non-parametric Kruskal-Wallis test was applied to the data (see Table 3) followed by the Dunn & Sidak post-hoc analysis (see Table 4). There was a statistically significant difference between groups, as determined by the Kruskal-Wallis test ($p=5.1043\times 10^{-14}$), and there is a significant difference between all treatments pairs, as one can observe by the results of the Dunn & Sidak post-hoc analysis. Extending the analysis, one can observe that, for all pairs, there is significant difference, hence the FSR-based approach can confidently be identified as the preferred approach for dynamic sound volume change.

*Task 4*

When users are required to move through a video into a desired time instant in YouTube, as done in the 4th task, considering only the average user preference level, the users preferred the FSR-based solution, with 2.73 average score. In this case, the PCAP-based slider was the second preferred option, with 1.67, while the capacitive buttons were the least preferred option, albeit with a close value, namely 1.6. An overview of the results is depicted in Figure 7. Following is an analysis that goes beyond the simple average user preference level assessment, i.e., analysis using significance tests. Although the homoscedastic condition is not violated, since the homogeneity ratio is 1.219, the Anderson-Darling test revealed that none of the treatment distribution was normal, hence violating the requirement for a parametric analysis. Therefore the non-parametric Kruskal-Wallis test was applied to the data (see Table 5), followed by the Dunn & Sidak post-hoc analysis (see Table 6). The $p=1.5264\times 10^{-8}$ is below the significance level of D = 0.05, therefore there is a statistically significant difference between groups. Given the results of the Dunn & Sidak post-hoc analysis, except for the pair But.Cap.-Sli.PCAP there is a significant difference between all other treatments pairs. Since the users preferred the FSR-based solution, and that all pairs with the FSR are significant different, one can conclude that differences in users sampled data did not happen by chance, and that they do reflect their preference for the FSR-based solution.

*Task 5*

Figure 8 shows the user preference average level concerning the evaluating of the various technologies for text input, gathered in the 5th task. Analysing the collected data one can verify that the homoscedastic condition test is not violated, since the homogeneity ratio is 1.699. The normality condition test revealed that none of the treatment distribution was normal, hence violating the requirement for a parametric analysis. Therefore the non-parametric Kruskal-Wallis test was applied to the data (see Table 7) followed by the Dunn & Sidak post-hoc analysis (see Table 8). There was a statistically significant difference between groups, as determined by the Kruskal-Wallis test ($p=3.7681\times 10^{-12}$), and there is a significant difference between all treatments pairs, as one can observe by the results of the Dunn & Sidak post-hoc analysis. Therefore, there are significant differences among interaction methods, meaning that one can proceed with the preferred technology analysis. As can be seen from Figure 8, most users preferred using the QWERTY keyboard for inputting text, resulting in 2.8 user preference average level. The second preferred option was the airmouse, with an average level of 1.93, while the least preferred option was the use of the trackpad (with the onscreen keyboard), with an average level of 1.27.

Furthermore, as shown in Table 9 and Figure 9, the average time required for each user to input the desired word was much lower when using the QWERTY keyboard (3.959 s), with the airmouse-based solution taking almost twice the time, and the trackpad-based solution taking more than three times that time. It is also important to notice that the text input time variation in the QWERTY case was also much lower that the other solutions, with the airmouse-based solution taking 13.473 s, and the trackpad-based solution taking 18.562 s, in the worst case.

### Comparison with Related Works-Discussion

Direct comparison of the above mentioned results with other works is not possible since the specific characteristics of the prototype were not replicated in any previously developed study, to the best of our knowledge. Nevertheless, it is possible to find related works with results that contribute to consolidate our findings. For instance, in [31] a user study was carried out to find the influence of haptic feedback on task-based presence and performance in virtual reality. Similar to our results, most users found haptic feedback to provide the greatest degree of presence and to improve the object detection rate (task performance). On the usefulness of haptic feedback, which is implicit in the results of this UX study, it was found in [32] that device-driven haptic feedback may lead to increase consumer responses to certain consumer-directed communications, by improving consumer performance on related tasks and an increased sense of social presence. A more generic study on influence of haptic feedback on emotional arousal, sense of presence, and embodiment in virtual reality, can be found in [33], where the main conclusions also corroborate those obtained in this work, i.e., more engaging experience is obtained when haptic feedback is used. The above cited works have in common with this one the fact that actual users participated in different evaluation studies and the main conclusions are coherent among them all, i.e., the use of haptic feedback is beneficial from different perspectives. This work further highlights system design elements and conditions that contribute to achieve consistent levels of UX.

## 5. Conclusions and Future Work

This work highlighted the need for remote control devices that maintain or improve the likability and usability of the traditional remote controls, while also allowing novel types of user interaction associated with rich multimedia content and Smart TV/Box. Different combinations of haptic feedback technologies which are ready to be mass-marketed in a remote control in the near future, were subjectively evaluated. This research took into account the emerging interaction needs required by Smart TV/Box and new types of multimedia content in user-centric contexts. Three main types of interactive actions were researched, namely, single-click (conventional) scenarios, dynamic inputs for actions like rapid sound volume change and video navigation, and tactile-based text input. It is also pointed out that LRA are much more efficient than ERM actuators, which is very relevant for this kind of battery-operated devices, where the same battery is expected to last for several months. The number of actuators is also a very important factor in terms of energy consumption. Fortunately, the user preference pointed towards such direction, given that the users have shown preference for using only one LRA actuator as opposed to using two LRA actuators. Regarding dynamic input tasks, the results show that FSR-based solutions clearly get the users preferences, allowing for a dynamic interaction that is not comparable with that of traditional mechanical buttons. Concerning text introduction, which is becoming more and more relevant for Smart TV/Box, the user preference average level and, particularly, the time results, demonstrated that having a QWERTY keyboard available to the user can greatly enhance their UX.

Overall the obtained results allow for important conclusions to be drawn, which provide useful guidelines for future research and engineering developments. Future work will be devoted to further research the user preferences of the haptic-based technologies addressed in this work, combined together with other technologies, such as the airmouse for point-and-click actions, or voice-based operations, as these are expected to be included in the forthcoming generation of remote controls. An interesting issue to be analysed in the future, is in terms of battery life when all these technologies are used extensively. Another open issue for further research is a possible dependency of the UX from the specific features of each actuator in addition to their type. For this purpose, different actuators of the same type must be subjectively evaluated to find possible variations in user preferences within each type.

## Figures and Tables

**Table 1:** Kruskal Wallis test for Task2.

	SS	df	MS	F	*p*
**Between groups**	85,140	3	28,380	75.0493	$3.5362\times 10^{-16}$
**Within groups**	49,860	116	429.8276		
**Total**	135,000	119			

**Table 2:** Dunn & Sidak Post-Hoc Analysis for Task2.

	${\bar{x}}_{i}-{\bar{x}}_{j}$	Confidence Lower	Interval Upper	Adjusted *p*-Values	Significance at 0.05
**ERM-LRA**	2.1190	25	47.8810	0.0240	YES
**ERM-PEA**	50.1190	73	95.8810	0	YES
**ERM-But.Mec.**	−0.8810	22	44.8810	0.0666	NO
**LRA-PEA**	25.1190	48	70.8810	$2.0407\times 10^{-7}$	YES
**LRA-But.Mec.**	−25.8810	−3	19.8810	0.9996	NO
**PEA-But.Mec.**	−73.8810	−51	−28.1190	$2.7048\times 10^{-8}$	YES

**Table 3:** Kruskal Wallis test for Task3.

	SS	df	MS	F	*p*
**Between groups**	37,140	2	18,570	61.2122	$5.1043\times 10^{-14}$
**Within groups**	16,860	87	193.7931		
**Total**	54,000	89			

**Table 4:** Dunn & Sidak Post-Hoc Analysis for Task3.

	${\bar{x}}_{i}-{\bar{x}}_{j}$	Confidence Lower	Interval Upper	Adjusted *p*-Values	Significance at 0.05
**FSR-But.Mec.**	16.8140	32	47.1860	$1.4603\times 10^{-6}$	YES
**FSR-Sli.PCAP**	33.8140	49	64.1860	$3.9302\times 10^{-14}$	YES
**But.Mec.-Sli.PCAP**	1.8140	17	32.1860	0.0224	YES

**Table 5:** Kruskal Wallis test for Task4.

	SS	df	MS	F	*p*
**Between groups**	21,840	2	10,920	35.9956	$1.5264\times 10^{-8}$
**Within groups**	32,160	87	369.6552		
**Total**	54,000	89			

**Table 6:** Dunn & Sidak Post-Hoc Analysis for Task4.

	${\bar{x}}_{i}-{\bar{x}}_{j}$	Confidence Lower	Interval Upper	Adjusted *p*-Values	Significance at 0.05
**FSR-But.Cap.**	18.8140	34	49.1860	$2.6987\times 10^{-7}$	YES
**FSR-Sli.PCAP**	16.8140	32	47.1860	$1.4603\times 10^{-6}$	YES
**But.Cap.-Sli.PCAP**	−17.1860	−2	13.1860	0.9850	NO

**Table 7:** Kruskal Wallis test for Task5.

	SS	df	MS	F	*p*
**Between groups**	31,920	2	15,960	52.6089	$3.7681\times 10^{-12}$
**Within groups**	22,080	87	253.7931		
**Total**	54,000	89			

**Table 8:** Dunn & Sidak Post-Hoc Analysis for Task5.

	${\bar{x}}_{i}-{\bar{x}}_{j}$	Confidence Lower	Interval Upper	Adjusted *p*-Values	Significance at 0.05
**Airmouse-Trackpad**	4.8140	20	35.1860	0.0050	YES
**Airmouse-QWERTY**	−41.1860	−26	−10.8140	$1.3049\times 10^{-4}$	YES
**Trackpad-QWERTY**	−61.1860	−46	−30.8140	$1.4202\times 10^{-12}$	YES

**Table 9:** Task 5-time needed for typing the word “ronaldo” using three different haptic technologies.

Technology	Average Introduction Time	Faster Introduction Time	Slower Introduction Time
Airmouse	10 s 882 ms	8 s 151 ms	13 s 473 ms
Trackpad	15 s 595 ms	13 s 317 ms	18 s 562 ms
QWERTY	3 s 959 ms	3 s 208 ms	4 s 580 ms

## Abbreviations

The following abbreviations are used in this manuscript:

ERM	Eccentric Rotating Mass
FSR	Force Sensing Resistor
HCI	Human Computer Interaction
IC	Integrated circuit
LRA	Linear Resonant Actuator
PCAP	Projected Capacity
PCB	Printed Circuit Board
PEA	PiezoElectric Actuator
PWM	Pulse Width Modulation
RCD	Remote Control Devices
UX	User eXperience
